# GastroPlus- and HSPiP-Oriented Predictive Parameters as the Basis of Valproic Acid-Loaded Mucoadhesive Cationic Nanoemulsion Gel for Improved Nose-to-Brain Delivery to Control Convulsion in Humans

**DOI:** 10.3390/gels9080603

**Published:** 2023-07-26

**Authors:** Afzal Hussain, Mohammad A. Altamimi, Mohhammad Ramzan, Mohd Aamir Mirza, Tahir Khuroo

**Affiliations:** 1 Department of Pharmaceutics, College of Pharmacy, King Saud University, Riyadh 11451, Saudi Arabia; maltamimi@ksu.edu.sa; 2 School of Pharmaceutical Sciences, Lovely Professional University, Phagwara 144411, India; mohhammad.26652@lpu.co.in; 3 Department of Pharmaceutics, School of Pharmaceutical Education and Research, Jamia Hamdard, New Delhi 110062, India; aamir.mirza@jamiahamdard.ac.in; 4 PGx Global Foundation, Houston, TX 77035, USA; tahir@pgxglobal.org

**Keywords:** valproic acid, GastroPlus-based prediction, cationic nanoemulsion, gels, in vitro–ex vivo permeation profile, CLSM study

## Abstract

Oral and parenteral delivery routes of valproic acid (VA) are associated with serious adverse effects, high hepatic metabolism, high clearance, and low bioavailability in the brain. A GastroPlus program was used to predict in vivo performance of immediate (IR) and sustained release (SR) products in humans. HSPiP software 5.4.08 predicted excipients with maximum possible miscibility of the drug. Based on the GastroPlus and HSPiP program, various excipients were screened for experimental solubility, nanoemulsions, and respective gel studies intended for nasal-to-brain delivery. These were characterized by size, size distribution, polydispersity index, zeta potential, morphology, pH, % transmittance, drug content, and viscosity. In vitro drug release, ex vivo permeation profile (goat nasal mucosa), and penetration studies were conducted. Results showed that in vivo oral drug dissolution and absorption were predicted as 98.6 mg and 18.8 mg, respectively, from both tablets (IR and SR) at 8 h using GastroPlus. The predicted drug access to the portal vein was substantially higher in IR (115 mg) compared to SR (82.6 mg). The plasma drug concentration–time profile predicted was in good agreement with published reports. The program predicted duodenum and jejunum as the prime sites of the drug absorption and no effect of nanonization on T_max_ for sustained release formulation. Hansen parameters suggested a suitable selection of excipients. The program recommended nasal-to-brain delivery of the drug using a cationic mucoadhesive nanoemulsion. The optimized CVE6 was associated with the optimal size (113 nm), low PDI (polydispersity index) (0.26), high zeta potential (+34.7 mV), high transmittance (97.8%), and high strength (0.7% *w*/*w*). In vitro release and ex vivo permeation of CVE6 were found to be substantially high as compared to anionic AVE6 and respective gels. A penetration study using confocal laser scanning microscopy (CLSM) executed high fluorescence intensity with CVE6 and CVE6-gel as compared to suspension and ANE6. This might be attributed to the electrostatic interaction existing between the mucosal membrane and nanoglobules. Thus, cationic nanoemulsions and respective mucoadhesive gels are promising strategies for the delivery of VA to the brain through intransal administration for the treatment of seizures and convulsions.

## 1. Introduction

Epilepsy is defined as a group of neurological issues of the central nervous system and is characterized as a predisposition to epileptic seizures due to the complexity of its characteristics. The World Health Organization (WHO) estimated that 50 million people are affected annually around the world [1]. In the USA, 2.3 million adults and 500,000 children are affected by varied forms of epilepsy due to unknown and known possible reasons (genetics, trauma, stroke, brain tumor, and any factors responsible for disturbing the normal pattern of the brain circuit) [2]. In Saudi Arabia, the reported prevalence of cases of epilepsy is 6.45 per 1000 people, which is responsible for affecting children’s mental health, behavior, and academic performance [3].

Valproic acid (VA) is the most effective first-line anticonvulsant to control grand mal epilepsy and tonic–clonic fits (seizure), various seizures, and idiopathic generalized seizures. Several characteristics, including low molecular weight (144 g/mol), hydrophobic nature (log P = 2.54), high oral dose (not more than 600 mg/kg/day), high first-pass metabolism (methylation, sulfation, and glucuronidation), and poor brain bioavailability after oral administration, are possible reasons for nasal delivery of the drug to control seizure [4]. Commercial products (oral and parenteral) showed high plasma levels of active metabolites (90% such as 4-ene-VA and undergoes beta oxidation of fatty acid) due to hepatic metabolism (causing hepatotoxicity) and rapid clearance due to efflux (P-gp pump of microvessel endothelial cell in the blood–brain barrier). Parenteral delivery causes serious side effects, possibly due to the reticuloendothelial system (Kuffer cells)-based metabolism and low bioavailability to the brain. Hammond et al. investigated the pharmacokinetics profiles of the drug in a cat model (six adult cats) after rapid intravenous infusion (60 mg/kg within 3 min of infusion using saline) wherein the maximum level of the drug was obtained at 1 min (brain distribution half-life as 6 min estimated from α-phase) followed by the Vd rapid clearance (mean elimination half-life of 41 min), and the volume of distribution (Vd) as 0.125 L/kg. Low in vivo uptake (low brain–plasma ratio), low Vd, and rapid clearance (brain elimination half-life of 41 min estimated from the β-phase) from the brain indicated poor binding of the drug to the cerebral cortex [5]. As per the US FDA label of DEPAKENE, oral absorption was dependent on age and dosage forms (tablet versus capsule). In adult patients, the absorption rate on monotherapy (250 mg of oral delivery) is nonlinear, whereas the kinetics of the unbound drug is linear. Notably, the drug is primarily metabolized through the liver (30–50% as glucuronide conjugate) and mitochondrial β-oxidation (>40%) for excretion through urine (3% as unchanged). In humans, the mean plasma clearance and Vd values were reported as 0.56 L/Kg and 11 L, respectively, following 250 mg of oral administration in adults (70 kg or 1.73 m^2^ as body surface area) [6].

Several drugs (35–40 molecules) have been exploited for brain delivery using the nasal route of administration. The route is the most preferred one to circumvent the aforementioned issues of oral and parenteral delivery in conventional dosage forms. Various nanocarriers have been reported for drug delivery through the nasal route. These are lipidic nanocarriers (lipid nanoparticles, nanoemulsion, and liposomes), nanotubes, and dendrimers [7,8,9,10,11]. Tan et al. tailored stable nanoemulsion comprised of safflower (70–80% linolenic acid-rich natural oil capable of drug delivery across the blood–brain barrier and cerebrospinal fluid barrier) for delivery of the drug to the brain, and brain bioavailability was improved [10,12].

Nanoemulsion is a well-explored nanocarrier system for drug delivery due to desired innate features such as nanoscale globular size, suitability to load small molecules, and thermodynamically stable isotropic mixture. An imposed cationic charge on the nanoglobule further improves its pharmaceutical utility for the facilitated permeation across the biological membrane for the extended residence time. Nasal drug delivery is usually challenged by its short residence time and high washout after nasal administration. Cationic-charged nanocarriers interact with the biological membrane for maximized internalization and increased passive permeation and drug deposition (enhanced drug access across the biological membrane) [13,14]. The nasal route of administration offers several advantages over oral and parenteral routes such as (a) high patient compliance, (b) avoiding hepatic metabolism and related drug degradation, (c) direct drug access from the olfactory region to the cranial cavity of the brain, (d) avoiding unnecessary administration of excipients, (e) dose mitigation and reduction in dose-related side effects, (f) low therapy cost, (g) ease in regulatory constraints for approval, and (h) safety and biocompatibility [14].

We predicted the in vivo performance of the drug using GastroPlus (predictive and simulation program) using the literature, default values, and experimental data. The program assisted in predicting the dose-dependent pharmacokinetic parameters (time required to reach C_max_ as T_max_, area under the curve as AUC, and maximum drug concentration reached in the blood as C_max_) considering an oral commercial dose (250 mg) and the dosage form (tablet) in healthy adults. Moreover, cationic nanoemulsions were prepared, optimized, and characterized for in vitro (size, size distribution, zeta potential, morphology, thermodynamic stability, and release profile at pH 5.5 and 6.8) and ex vivo performance (permeation flux, drug deposition, and enhancement ratio) (goat nasal tissue).

## 2. Results and Discussion

### 2.1. Prediction and Simulation Study Using GastroPlus

**Figure 1:**
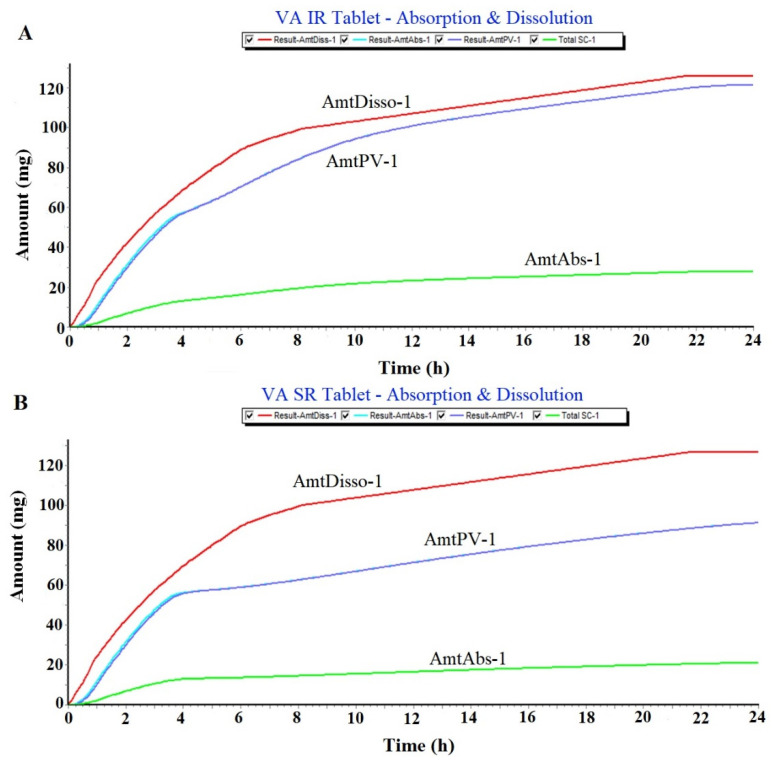
Simulation and prediction software (GastroPlus)-based analysis of IR tablet and SR tablet for oral administration. (**A**) Prediction of in vivo dissolution and in vivo absorption of VA IR Tablet (200 mg) for oral delivery (once a day in fast-state condition) and (**B**) prediction of in vivo dissolution and in vivo absorption of VA SR tablet (200 mg) for oral delivery (once a day in fast-state condition).

VA is orally administered in different dosage forms such as IR/SR tablets, an oral solution, and an oral capsule. Considering 200 mg as an adult dose in an IR tablet and SR tablet, the program was run for a simulation of 24 h (Table 1). The program was used to predict PK parameters (C_max_, AUC, and T_max_) for both of them in adults. Limited data are available for comparative PK studies of VA using IR and SR tablets in humans. No data are available for predicting PK parameters using the GastroPlus program and comparing an IR tablet and an SR tablet at a fixed dose and dosing frequency. Teixeira-da-Silva et al. predicted population pharmacokinetics of VA monotherapy considering different doses, body weights, and age groups. The regimen depicted was designed to achieve a VA concentration within the acceptable therapeutic range. The steady-state plasma concentrations were predicted to be >120 mg/L for age groups of 15 (1000 mg in tablet) and 35 years (1200 mg in tablet), whereas this value was predicted as <100 mg/L for children aged 1 (dose of 100 mg in solution) and 6 years (dose of 200 mg in solution) [15]. Thus, the authors found that there was no significant difference in the plasma drug concentration from the tablet with 1000 mg or 1200 mg in adults of different ages (15 versus 35 years old) and body weights (56 versus 70 kg) [16]. In the present study, we used a 200 mg dose for an adult weighing 70 kg to predict in vivo dissolution and in vivo absorption of the IR tablet and SR tablet of VA. The result is illustrated in Figure 1A,B. It is clear that the predicted pattern of in vivo dissolution of the IR VA tablet and the SR VA tablet are closely related without a substantial difference in a fast-state adult. Interestingly, the amount of drug absorbed by the portal vein (AmtPV-1) is higher in the IR VA tablet compared to the SR VA tablet (Figure 1A) as predicted in the program. This may be prudent to correlate the difference in the dissolution rate between IR and SR tablets. The IR tablet exhibits rapid drug dissolution in gastric content for the profound availability of the drug for absorption at the intestinal mucosa of the GIT lumen. On the other hand, the SR tablet follows a different dissolution process due to the rate-limiting membrane of the polymer coating on the tablet. Slow and sustained release of the drug caused slow and extended absorption as predicted in Figure 1B. The total amount of the drug absorbed from both tablets is approximately the same as predicted in the program (green bold color) suggesting no significant difference in the modified form of the tablet over a period of 24 h. This may be due to the slightly acidic nature of VA (pKa = 5.14) suitable for absorption from the intestinal area as the prime site of drug absorption. The therapeutic window of the drug is 30–100 mg/L after oral administration in humans [17]. The drug is rapidly absorbed from the oral dosage form and the drug access to the brain is limited due to the high protein binding capacity (90%). The low volume of distribution (0.125 L/Kg) is very similar to that found in humans suggesting no significant bounding of the drug to the brain. Therefore, this needs a high blood plasma level by administering high oral doses. The limited free drug in the plasma is available for brain access. In a previous study, it was observed that VA transport to the brain occurs via the monocarboxylic acid transport system. The plasma level of VA < 60 µg/mL results in a low level of the drug in the brain. For clinical effectiveness in humans, it is only possible with a relatively high plasma concentration above 55 µg/mL [5].

#### 2.1.1. Prediction of Plasma Drug Concentration Time Profile

**Figure 2:**
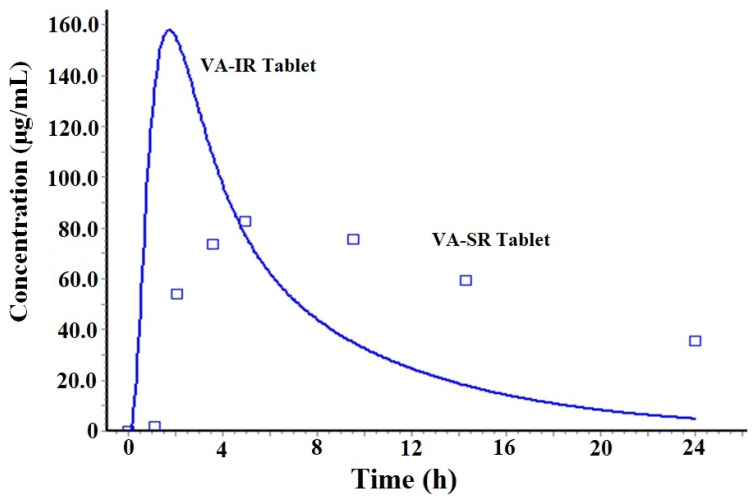
Plasma drug concentration–time profile predicted for VA IR tablet and VA SR tablet (200 mg). This was a predicted profile of a human in fast-state condition using GastroPlus.

The program predicted the plasma drug concentration–time profile of the IR tablet and the SR tablet of VA. The result of the predicted PK profile is displayed in Figure 2 wherein C_max_ values of VA IR and VA SR tablets were predicted as 159.3 µg/mL and 82.5 µg/mL, respectively. The predicted values are quite interesting and convincing as explained before for therapeutic effectiveness. Both values are enough to produce a substantial level of the drug in the blood plasma for brain access (>55 µg/mL) [5]. The acidic form of the drug is suitable for solubility in water and an acidic medium (pKa 5.4). Therefore, the IR tablet showed rapid drug dissolution for immediate drug absorption. Therefore, the IR tablet (2.1 h) showed relatively low T_max_ compared to the SR tablet (5.2 h) in prediction. These predicted T_max_ values are in good agreement with the published report for the oral solution and the SR tablet [18]. This indicated that the model is a good fit (as observed by the high Akaike value) for simulation and prediction. The result can be correlated to the difference in oral bioavailability of VA in the drug solution and the SR formulation. In the literature, the drug solution and SR formulation resulted in 100% and 80–90% bioavailability for VA [19]. Thus, the predicted pattern of the VA SR tablet suggested slow and sustained delivery of VA for a long-term effect within the therapeutic window (200 mg). However, the drug is limited to brain access due to various possible reasons. These may be due to high protein binding capacity, high hepatic drug metabolism, the low solubility of the drug, and extra hepatic drug metabolism. The sustained-release tablet slightly decreased the drug absorption to the portal vein (Figure 1B).

#### 2.1.2. Regional Compartmental Absorption of Both Tablets

**Figure 3:**
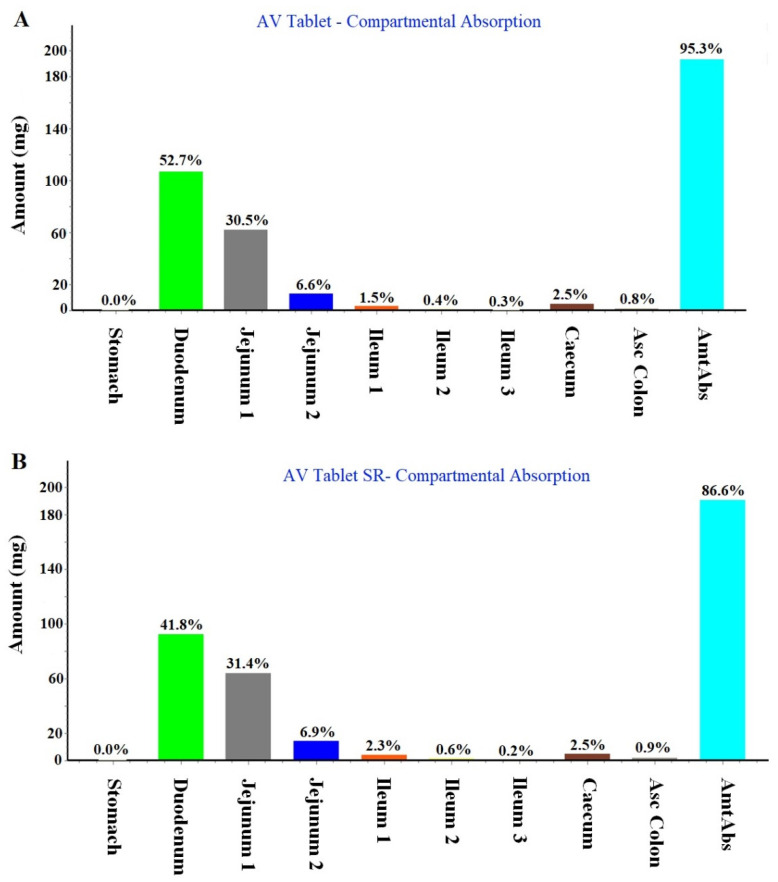
Regional compartmental absorption of the drug from human GIT (gastrointestinal tract) in fasted condition: (**A**) Prediction for the IR tablet and (**B**) predicted values for SR tablet.

The program predicted nine compartmental absorption sites in GIT. Both IR and SR tablets were processed in the system to estimate the percent of regional absorption of the drug. The result is displayed in Figure 3A,B wherein the IR tablet and the SR tablet were predicted to have an overall total absorption of 95.3% and 86.6%, respectively. VA is a slightly acidic drug with a pKa value of 5.6. Therefore, the drug was predicted to be absorbed primarily from the proximal portion of GIT. Thus, the duodenum and jejunum are the main sites of oral absorption. The drug is considered poorly absorbed from the distal GIT region as shown in Figure 3A,B. The predicted values are in agreement with the published report of oral bioavailability for the drug solution (100%) and SR tablet (80–90%) [19].

#### 2.1.3. PSA (Parameter Sensitivity Analysis) Assessment

**Figure 4:**
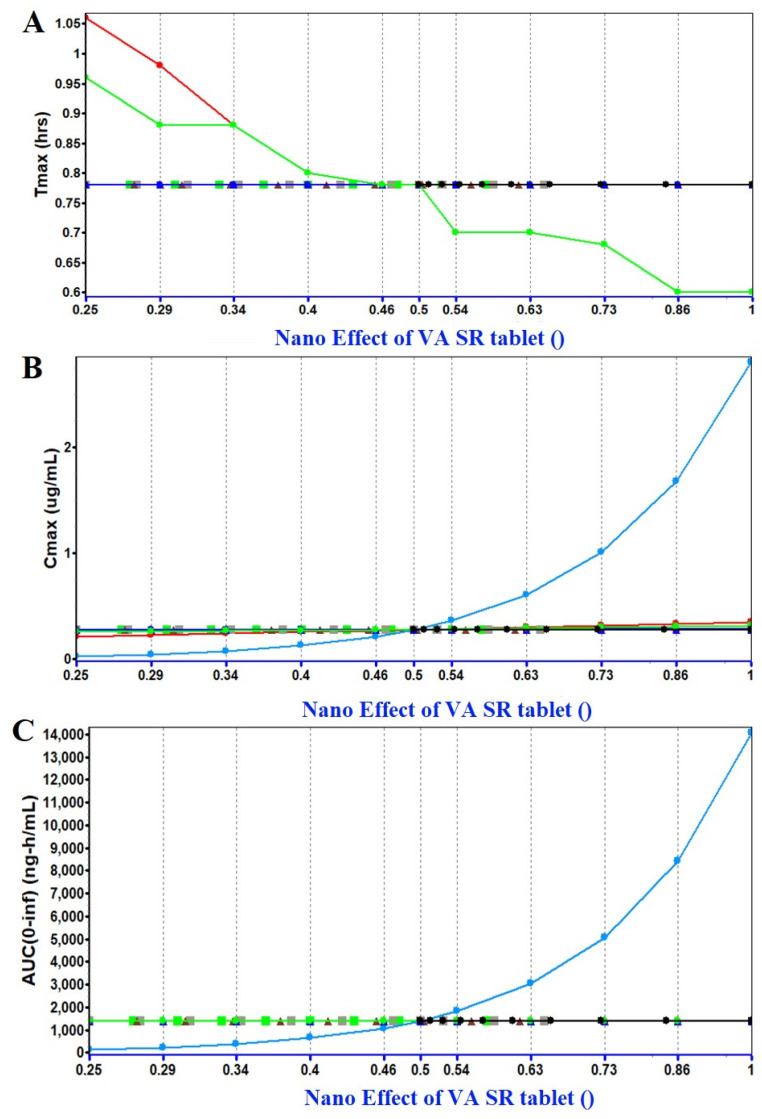
Parameter sensitivity analysis assessment using GastroPlus Version 9.8.3 simulation and prediction program. (**A**) Impact of nanonized VA product (oral delivery) on T_max_, (**B**) impact of nanonized product on C_max_, and (**C**) impact of nanonized product on AUC values. Blue bold line indicates the impact of Nano effect. Red bold line indicates “P_eff_” permeability coefficient across mucosal membrane, and effect of duodenum ASF (absorption scale factor). The green curve indicates the impact of reference solubility. Sky blue curve indicates the dose effect. The brown triangle symbol represents particle density whereas grey curve indicated shape behaviour.

PSA assessment was performed to identify the relevant factors responsible for affecting the PK parameters of the VA tablet on oral administration (Figure 4A–C). The analysis was carried out using the GastroPlus program considering the fasted-state condition of the subject. This avoided any interaction with food. In the study, we attempted to predict the impact of a nanocarrier system for oral drug delivery and its impact on PK parameters such as C_max_, T_max_, and AUC (area under the curve). It is clear from the prediction study and literature-based findings that conventional dosage forms of VA (tablet, SR tablet, and solution) do not have much difference in terms of bioavailability in humans [10,16,20,21,22,23,24]. Therefore, the predicted oral bioavailability values are almost similar to the reported values (as described before). However, the program predicted that the nano effect had no impact on PK parameters after oral delivery. This may be correlated with the lipophilic nature of the drug being absorbed and poor dissolution (BCS class II) [25,26]. Conclusively, GastroPlus simulation and the prediction program assisted in the understanding of the nanonized product of VA for oral delivery could be of no benefit for brain delivery. Therefore, it is better to formulate nanocarrier-based drug delivery for brain delivery using the nasal route of administration. The basal route contains the olfactory chamber directly linked to the brain for drug access. Thus, the purpose of GastroPlus-based prediction was to understand the feasibility of the oral nanocarrier for brain delivery using clinical data (obtained from the literature). The program provided various predicted in vivo values for a human trial.

#### 2.1.4. Hansen Solubility Parameters for VA and Excipients

HSP values helped to select excipients possibly exhibiting maximum drug solubility via a cohesive interaction (cohesive forces) [27]. The program is well-exploited for solute miscibility/solubility in a particular solvent. The HSP values of the drug and each excipient are summarized in Table 2. It is easy to understand that the lipid, the surfactant, and the co-surfactant possessing HSP close to the values of the drug could be the most appropriate and suitable for drug solubility. The values of δ_d_, δ_p_, and δ_h_ of the drug are 16.1, 4.3, and 9.0 MPa^1/2^, respectively. The δ_h_ value of tween 80 is quite close to the HSP values of VA compared to span 80 (δ_h_ of span 80 is 12.4 compared to 9 of tween 80). Therefore, a solute interacts with a solvent through these cohesive forces working together. Thus, the difference of any parameter between the solute and solvent close to zero is considered miscible or soluble. Thus, the program predicted relevant excipients based on these HSP values of each excipient close to the HSP values of the model drug. The program estimated these values as shown in Table 2. The HSP values of oils (safflower, Flaxseed oil, and grape seed oil), lecithin, and PC were obtained from the literature and calculated manually based on the percent composition of linoleic acid or phosphatidylcholine (PC) present [28]. Among these oils, safflower seed oil might be the most suitable for tailoring a cationic nanoemulsion due to the predicted miscibility of the drug in terms of HSP. The oil has been reported t have a high content of linoleic acid (78%), and linoleic acid is considered a promoter for the diffusion of the lipophilic drug across the blood–brain barrier [29].

#### 2.1.5. Solubility of Valproate (VA) in Various Excipients

**Figure 5:**
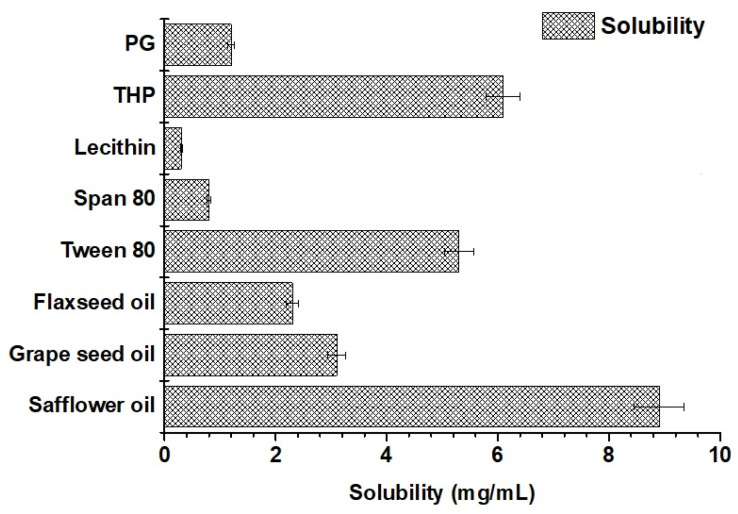
Experimental solubility of VA in various excipients at 40 °C. Data were expressed as mean ± standard deviation (*n* = 3).

The result of the experimental solubility of VA is portrayed in Figure 5. The solubility of the drug was found to be the maximum in safflower seed oil (8.9 ± 0.11 mg/mL), tween 80 (5.3 ± 0.09 mg/mL), and transcutol (6.3 ± 0.08 mg/mL). This maximized solubility can be rationalized based on the HSP values predicted in Table 2. The difference value of Δδ_d_ is 1.6 (16.1–14.5) for the solute (VA) and the solvent (safflower oil), which is quite low for high miscibility/solubility. Similarly, the difference values of Δδ_p_ and Δδ_h_ are 1.6 (4.3–2.7) and 3.7 (9–5.3), respectively, for the drug in safflower. These differences are quite convincing for maximized drug solubility due to interactive forces (polarization, hydrogen bonding ability, and dispersion nature). Among the co-surfactants, transcutol was selected due to the highest solubility and suitability for the drug. Flaxseed (50–70%), safflower (70–87%), and grape seed oil (70%) are prime sources of linoleic acid. Linoleic acid-rich oils are gaining popularity in pharmaceutical and cosmeceutical industries due to possessing various skin benefits such as (a) anti-inflammatory, (b) acne-reductive, (c) skin-softening, and (d) moisture-retentive qualities, as well as possessing the ability to (e) facilitate drug diffusion across the blood–brain barrier (50–87% linoleic acid) and (f) biocompatibility [10,31]. Thus, safflower, tween 80, and transcutol were selected as the oil, surfactant, and co-surfactant. However, a blend of tween 80 and lecithin (1:1) was used for a stable and small-sized nanoemulsion. The combination was supposed to stabilize the nanoemulsion with a small size particle as compared to tween 80 as a standalone.

#### 2.1.6. VA Loaded Cationic Nanoemulsions Prepared

To construct a cationic nanoemulsion, a constant amount of stearylamine (5 mg) was used in each formulation. A series of nanoemulsions (CVE as cationic and AVE as anionic nanoemulsion) were prepared as shown in Table 3. Formulation CVE5 exhibited unique characteristic features among them. The globular size, PDI, zeta potential, %T, and product strength (% *w*/*w*) were found to be 79 nm (the lowest value), 0.11 (the lowest value), +27.1 mV (optimal), 95%, and 0.5%, respectively. The lowest value of PDI is due to the lowest content of oil (9.8%) and the sufficient amount of S_mix_ (21.84%) responsible for efficient emulsification and resulted in the homogeneous nature of the globular distribution. However, %DC was found to be low (~0.5%) for CVE5, which may be related to the low content of oil (9.8%). Formulation CVE6 had an optimal content of oil (14.46%) and S_mix_ (17.15%) to render the optimal size (113 nm), high zeta potential (+34.7 mV) for enhanced stability, and high %DC (67%) as compared to others. Comparing CVE1, CVE3, and CVE4, it is clear that by increasing the relative concentration of S_mix_ compared to oil, the size values regularly decreased from 185 nm to 148 nm. This may be due to efficient emulsification by the surfactant mixture. Comparing CVE2 (189 nm) and CVE4 (148 nm), the size of CVE4 was substantially decreased due to the high content of the surfactant mixture, even decreasing the relative content of the co-surfactant transcutol (from 1:3 to 1:2) within the S_mix_. AVE6 was anionic (zeta potential = −22 mV) in nature due to the lack of stearylamine in the formulation and served as a control group. The negative zeta potential is due to the lipid (triglycerides of fatty acids). The study aimed to address the impact of the charge on the nanocarrier for permeation behavior across nasal mucosa followed by blood–brain barrier. Notably, all of the nanoemulsions showed %T (%transmittance) higher than 96% suggesting the isotropic and transparent nature of the cationic and anionic nanoemulsions.

#### 2.1.7. Freeze–Thaw Cycle and Ultracentrifugation of Nanoemulsions

The developed formulations CVE1-CVE6 and AVE6 were subjected to ensure stability and capability to withstand thermal and physical stress during storage and transportation. The centrifugation step confirmed the physical stability to face attrition- and friction-triggered phase separation usually observed during transportation [32]. On the other hand, extreme temperatures (freeze and accelerated temperatures) assured stability against thermal-mediated instability in the nanoemulsion. The result is presented in Table 4. All of the formulations (cationic and anionic) were physically and thermally stable at the explored temperatures for the studied time period. A sequential series of thermal exposure from low to high via room temperature indicated that each product resumed its original state of the transparent isotropic nature of the nanoemulsion with good flowability, consistency, and elegancy. There were no signs of any instability over the explored period of time. It was imperative to corroborate the thermal and physical stability so that the developed nanoemulsion can be stored and transported accordingly.

### 2.2. Evaluation of Cationic and Anionic Nanoemulsions Gels

CVE6 and AVE6 were used to incorporate 1% carbopol gel (1:1 ratio) into the respective nanoemulsion gel (0.5%) containing 0.35% *w*/*w* of VA in the gels. Thus, the final product strength was 0.35% *w*/*w* in each gel. Both gels were evaluated for size, PDI, ZP, viscosity, and final pH as shown in Table 3. It is apparent that the pH (from 7.4 to 6.8) and zeta potential (from +34.7 to +21.9 mV) values of the CVE6 gel were significantly reduced from the respective CVE6 nanoemulsion. This is obvious due to the acidic carbopol polymeric gel with free carboxylic acid in its structural backbone. However, globular size values were nearly similar to the CVE6 nanoemulsion, suggesting no globular aggregation in the gel carrier. Viscosity values of the CVE6 gel and the AVE6 gel were 1837 and 1907 cP, respectively. These findings are in good agreement with the reported 0.5% carbopol 934 gel for topical application [33]. The viscosity indicates good consistency and shear thinning behavior after topical application due to the oil in a water-based system. In the final selected formulations, a fixed amount of SA was used in CVE6 and CVE6-gel to achieve concerted positivity on the globular surface of the nanoemulsion, which may facilitate the mucoadhesive property (as a result of electrostatic interaction) after nasal administration to improve the residence time and absorption [34]. The final pH of CVE6, AVE6, CVE6-gel, and AVE-6 gel products was found to be in the range of 6.8–7.4, which provided agreeable consistency and compatibility with the nasal mucosa.

#### 2.2.1. Morphological Evaluation of the Optimized Cationic Nanoemulsion and Respective Gel

CVE6, AVE6, CVE6-gel, and AVE6-gel were considered the most stable and optimized nanoemulsion and respective gels. Generally, the size, shape, and size distribution are expected to change after the incorporation of the nanoemulsion into a hydrogel carrier. Therefore, it was requisite to visualize CVE6, AVE6, CVE6-gel, and AVE6-gel. Thus, the morphology of nanoemulsions was compared after incorporation into the gel. The result is shown in Supplementary Figure S1 including the shape, size, and globular size distribution. The shape of the globular particle is approximately similar in the nanoemulsion and respective gel. However, the cationic nanoemulsion is found to be well dispersed in CVE6 compared to AVE6, which may be due to the imposed positive charge. AVE6 is slightly dispersed without forming any aggregation. A similar observation was obtained in the respective gel. Thus, hydrogel could not change the shape, size, or globular distribution of the nanoemulsion. Moreover, there was no observed drug precipitation even after the amalgamation of CVE6 or AVE6 into the carbopol hydrogel matrix. This suggested there was a substantially firm layer of S_mix_ coated on oil globules containing solubilized VA. It is noteworthy that the size obtained from DLS always differs from the size estimated using TEM. This happens due to instrumental error and differences in the working principle. Both techniques are quite different and followed different sample processes during analysis. Therefore, this error is defined as a “Fold error” and estimated using the following formula:

Fold error (FE) = 1/n [log^size of DLS/size of TEM^] (1)

In general, the error is considered acceptable when it drops below 2 (≤2) [35]. The values of FE for CVE6, AVE6, CVE6-gel, and AVE6 gel were found to be 1.4, 1.7, 1.3, and 1.9, respectively. For the gel, the sample was first diluted in water to a gel consistency similar to the respective nanoemulsion before analysis using the DLS technique. The same sample was scanned under TEM. In DLS analysis and TEM-based scanning, the temperature was kept constant to avoid any further errors in the results.

#### 2.2.2. Drug Content Estimation

The percent drug contents of CVE1-CVE6, AVE6, AVE6-gel, and CVE6 gel were estimated using the HPLC method. The sample was dissolved in an acetonitrile-methanol mixture (30:70) to extract the drug. The sample was filtered and analyzed. The percent of drug content in each formulation was not less than 99.3%. There was a slight loss of drug content during the preparation and handling process. The percent strength of each nanoemulsion and gel is presented in Table 3.

#### 2.2.3. In Vitro Drug Release Profile

**Figure 6:**
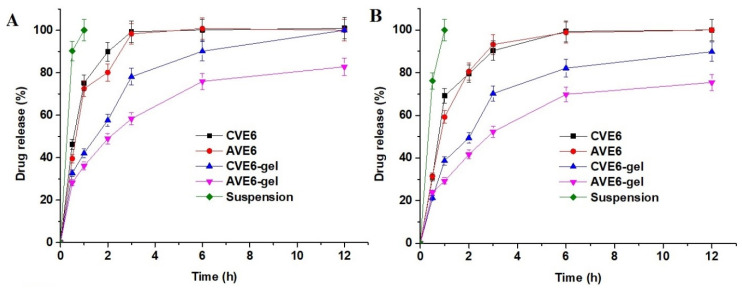
In vitro drug release using a dialysis membrane: (**A**) The drug release profile at pH 6.8 and (**B**) the drug release profile at pH 7.4 and 37 ± 1 °C (data are expressed as mean ± standard deviation, *n* = 3).

The model drug is acidic in nature (pKa = 5.2) and poorly soluble in water (1.3 mg/mL). The drug is reported to be soluble in an alkaline medium such as sodium hydroxide and alcohol. The optimized nanoemulsions (CVE6 and AVE6) and their respective gels showed different release behavior at pH 6.8 and 7.4 (phosphate buffer solution). The result is presented in Figure 6A,B. The nasal fluid and mucosa pH is approximately 6.8 and systemic delivery across the blood–brain barrier is exposed to pH of 7.4. Therefore, it was mandatory to investigate the impact of mucosal pH and blood pH when formulations are expected to be transported across mucosal and BBB for brain delivery. The result showed two important findings. These were (a) the impact of gel and (b) the impact of the release medium pH. It is quite clear that the drug was rapidly released from cationic and anionic nanoemulsions through the dialysis membrane as compared to the respective gel. This may be correlated with the viscous nature of the gel and two drug-release-limiting factors. These drug release rate-limiting factors are the gel matrix and dialysis membrane slowing down nanoglobules diffusion from the matrix to the medium. In the case of the nanoemulsion, there is only the dialysis membrane as a drug-release rate-limiting factor. The low viscosity further facilitated drug diffusion from the nanoemulsion to the release medium [36]. The release medium chamber was maintained at a temperature of 32 ± 1 °C throughout the study. The drug suspension (7 mg/mL) was rapidly released (>90%) within 30 min due to its salt solubility (1.3 mg/mL) at pH 6.8 (Figure 6A). A similar pattern was observed at pH 7.4 (>78.4% within 30) (Figure 6B).

#### 2.2.4. Ex Vivo Drug Permeation and Drug Deposition Using Goat Nasal Mucosal Tissue

**Figure 7:**
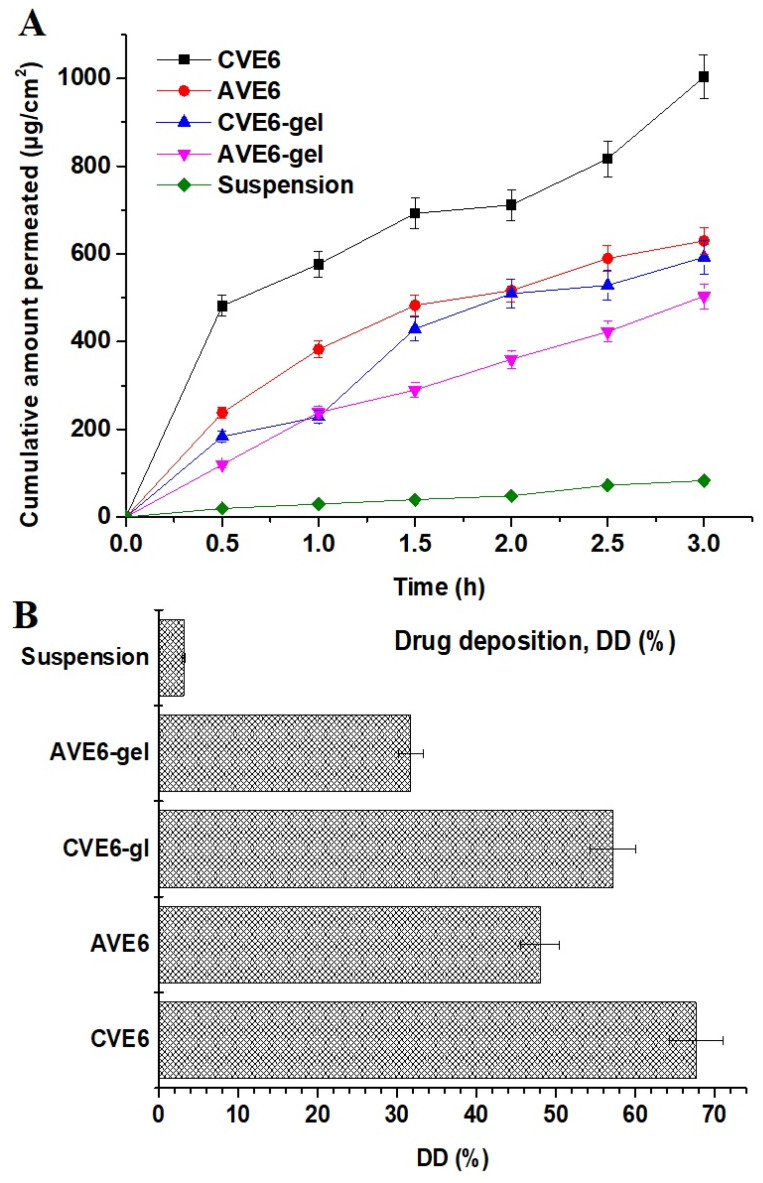
(**A**) Ex vivo cumulative drug permeation across nasal mucosa of goat over a period of 6 h in simulated nasal fluid, and (**B**) drug deposition of the drug in nasal mucosa after 6 h of ex vivo permeation at 37 ± 1 °C (data are expressed as mean ± standard deviation, *n* = 3).

Various reports have been published for drug delivery to the brain using the nasal route. The nasal mucosa composition, the viscosity of the nasal formulation, mucoadhesiveness, residence time, and nasal pH are major critical factors responsible for controlled drug release and permeation across the nasal epithelium [37,38]. The study was conducted for up to 6 h to avoid any loss of natural anatomical structural integrity of mucosal tissue and tissue viability [39]. The study was conducted using a simulated nasal fluid with pH 6.8 (to mimic nasal pH) to avoid nasal irritation and discomfort after application [40]. Gel products are relatively viscous and more mucoadhesive compared to CVE6 and AVE6. The cumulative amount of drug permeation is revealed in Figure 7A and the drug deposition into the nasal mucosal tissue is presented in Figure 7B. The values of permeation flux for CVE6, AVE6, CVE6-gel, and AVE6-gel were estimated as 67.64, 48. 01, 57.18, 31.74, and 3.15 µg/cm^2^/h, respectively, across the nasal mucosa of goats. The steady-state permeation flux values of the cationic nanoemulsion and its gel exhibited 21.47- and 18.15-fold higher flux rates as compared to the control suspension, which may be correlated with cationic and mucoadhesive gel carriers providing an electrostatic interaction with a negatively charged mucosal surface, extended residence time, and linoleic acid reported to facilitate drug permeation across the blood–brain barrier [41,42]. Moreover, the gel is mucoadhesive, biocompatible, and slightly acidic comparable to nasal fluid pH and drug pKa value (5.2–5.6). The flux value of CVE6 is very comparable to the published report of flux (~73 µg/cm^2^/h) for a VA-loaded niosomal in situ gel across a goat mucosal membrane [43]. Slightly high flux values may be attributed to a niosomal loading efficiency greater than the nanoemulsion. Fortunately, these parameters are suitable for maximized nasal permeation of the drug in the explored carrier for brain delivery. The drug is supposed to remain unionized at nasal pH due to the comparable pKa value for enhanced permeation and drug deposition. In addition, considering poorly vascularized (anterior third of each nasal cavity) and highly vascularized anatomical areas (the respiratory epithelium and two-thirds of the posterior portion of the cavity) of the nose, inhaled particles or nanoglobules were thought to be lodged by three prime mechanisms, namely (a) gravitational sedimentation, (b) inertial impaction, and (c) Brownian diffusion (if spayed) [43]. To understand the mechanistic perspective of drug delivery from the nose to the brain, it is imperative to consider the interplay of various critical factors such as formulation characteristics, the device, and patient-related conditions. These factors are directly involved in the drug-laden nanodroplets for maximized permeation and drug deposition within nasal cavities and, subsequently, the drug access to the brain. Notably, the exact localization of the drug for deposition is recognized as key to the success or failure of the nasal product [44]. The sites of drug localization within the nose dictate the purpose of local, systemic, and brain drug delivery. For drug delivery to the brain, the nasal cavities (innervated with olfactory and trigeminal nerves) are the most ideal site for drug localization and constitute a potential target for nose-to-brain delivery using a cationic nanoemulsion and gel formulation. Moreover, these cavities rapidly absorb the lodged drug through the thin membrane to achieve faster onset of action at a low dose, high patient compliance, reduced dose and metabolite (4-eve-VPA)-based side effects (hepatic toxicity due to the reticuloendothelial system) without hepatic metabolism and maximized drug access to the brain [45,46,47]. Greater uptake by RES indicates greater drug metabolism and incidence of side effects. Considering formulation-related factors such as the globular size, shape, zeta potential, viscosity, and mucoadhesiveness, the drug solubility, polarity, hydrophilicity, and composition (surfactant and oil) are complementary factors. Linoleic acid-rich oils are gaining popularity in pharmaceutical and cosmeceutical industries due to possessing various skin benefits such as (a) anti-inflammatory, (b) acne-reductive, (c) skin-softening, and (d) moisture-retentive abilities, as well as (e) facilitating drug diffusion across the blood–brain barrier (50–70% linoleic acid) and (f) biocompatibility [10,31]. Tween 80 possessed high hydrophilicity due to the high HLB value (14.5) and it is anticipated to achieve maximized emulsification in the hydrophilic mucosal layer to keep nanoglobules in an emulsified form within the mucosal matrix for prolonged systemic circulation time (likely due to the long fatty acid chain in lipid. such as linoleic acid) in the brain or reduced RES uptake. The surfactant is reported to have several benefits for nasal nanoemulsion for VA delivery to the brain. These are (a) protection of the drug from enzymatic degradation, (b) improved brain bioavailability, and (c) prolonged circulation time in the brain due to the long fatty acid and polyunsaturated fatty acid (PUFA) of the present oil [10].

The result of the drug deposition is presented in Figure 7B wherein CVE6, AVE6, CVE6-gel, AVE6-gel, and the suspension showed percent drug depositions of 67.64, 48.0, 57.18, 31.74, and 3.15%, respectively. It is quite clear that greater drug deposition means greater permeation flux as observed in CVE-6 as compared to the respective gel and other nanoemulsions. The gel matrix slightly delayed permeation and drug deposition, which is good for prolonged drug release and an extended effect to control epileptic fits and seizures. However, considering the types of patients and working or traveling schedules, both formulations are important. For immediate relief, it is better to spray a cationic nanoemulsion as it is aqueous and free-flowing due to its low viscosity. In the case of a planned traveling schedule, a gel product is better and more suitable as a prophylactic dose for prolonged relief from seizure attacks. Globular size, surface charge, and pH are other factors controlling drug deposition and, subsequently, drug flux. The nanoemulsion size depends on the oil content (the oil content is inversely proportional to the globular size of the nanoemulsion) and the content and type of surfactant. Tan et al. revealed reduced globular sizes of the nanoemulsion from 142 nm to 80 nm due to the reduced content of oil from 6% to 1.5%, respectively [10]. In the literature, it was reported that VA transport and nanoemulsion permeation across the blood–brain barrier is mediated via the organic anion transporter and the LDL-mediated endocytosis due to the presence of tween 80, respectively [48,49,50]. This may explain the significant difference in permeation profiles between the drug suspension and formulations.

#### 2.2.5. Confocal Laser Scanning Microscopy (CLSM)

**Figure 8:**
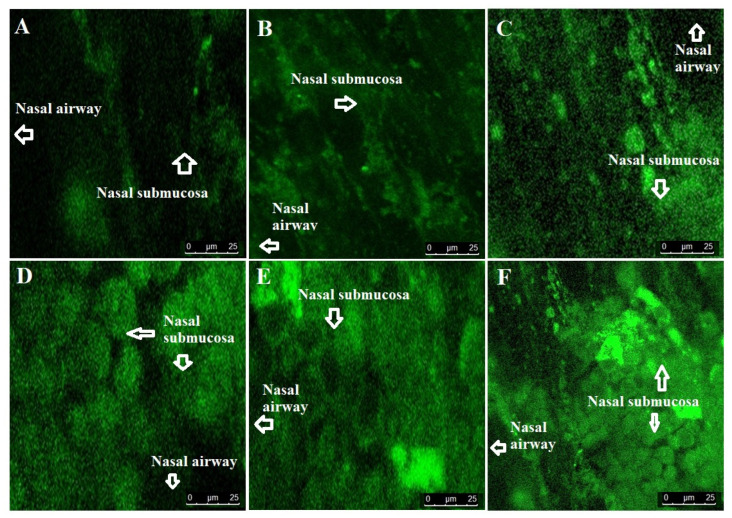
Penetration and permeation of the optimized nanoemulsions and respective gels across nasal epithelium to submucosal and mucosal regions using CLSM (confocal laser scanning microscopy). (**A**) Control using R123 solution, (**B**) R123-probed drug suspension, (**C**) AVE6-R nanoemulsion, (**D**) AVE6-R-gel, (**E**) CVE6-R nanoemulsion, and (**F**) CVE6-R-gel. Mean intensity measured using image J software 1.54f (**E**).

To evaluate the degree of penetration and permeation across the superior nasal concha (nasal membrane), we scanned the nasal mucosa treated with the formulations under CLSM. For comparison, the R123 solution was used as the control. The result is provided in Figure 8A–F. It is obvious from the result that the dye solution and suspension were not penetrable across the hydrophilic (approximately 90–95% water and glycoprotein, providing a gel-like structure) nasal mucosal membrane as evidenced by the poor fluorescence intensity [51]. The drug suspension containing the dye showed approximately similar intensity due to the drug insolubility and poor permeation behavior. The fluorescence intensity values of the dye solution, suspension, AVE6-R, AVE6-R-gel, CVE6-R, and CVE6-R-gel were obtained as 11.6, 17.3, 65.6, 75.62, 84.7, and 96.11%, respectively. The lowest fluorescence intensity associated with the dye solution and suspension could be attributed to poor dye and drug permeation across the hydrophilic nasal mucosa as a result of low solubility. However, high-intensity values were observed for both the nanoemulsions (AVE6-R and CVE6-R) and gels (AVE6-R-gel and CVE6-R-gel) as shown in Figure 8. A high degree of intense fluorescence by the gel and cationic nanoemulsion can be correlated with mucoadhesiveness and prolonged residence time on the nasal mucosa of goats. Carbopol gel is known for its good mucoadhesive nature at compatible pH for nasal delivery (4.5–6.8) without producing any nasal irritation [52]. Nasal pH (4.5–6.8) is very suitable for the gel consistency maintained after nasal application. Moreover, the drug is slightly acidic to ensure it is in a stable and non-ionized form if it comes into contact with the nasal fluid and mucosal membrane. The drug- and formulation-related properties provide suitability for the drug permeation, penetration, and compatibility for intranasal delivery of the drug to control convulsion in patients. Moreover, the imposed positive charge on the cationic nanoemulsion facilitated the nanoemulsion penetration as compared to the anionic counterpart as evidenced by the remarkably high fluorescence intensity. This can be correlated to the electrostatic interaction-mediated improved permeation and, subsequently, the drug deposition within the submucosal region of nasal tissues. In addition, intranasal delivery of the nanocarrier-based drug offers several advantages over oral administration of the drug.

Conclusively, the dye solution and the drug suspension itself are not capable of being penetrated. Both nanoemulsions were relatively less viscous as compared to the gel formulation. This caused slightly lower residence time in the mucosal region. The gel carrier provided hydration and high residence time for nanoemulsion penetration. Finally, the cationic globular electrostatic interaction with the negatively charged nasal membrane rendered the investigated nanoemulsion suitable for maximized permeation and penetration [53]. Thus, it was hypothesized that the optimized viscosity, imposed cationic charge, reduced globular size, and mucoadhesive gel could be working in tandem for drug delivery to the brain through nasal administration.

## 3. Conclusions

The conventional dosage form of VA is associated with multiple challenges. These challenges are related to the physicochemical properties, pharmacokinetic behavior, and pharmacodynamics properties of the drug. Low bioavailability to the brain, high hepatic metabolism, and severe side effects upon oral and parenteral delivery gained widespread attention from formulation scientists for alternative and high therapeutic benefits. The GastroPlus program assisted us to understand the in vivo behavior of the drug in the human body at the explored dose, dosing frequency, and dosage form. Moreover, the program predicted various factors responsible for affecting in vivo pharmacokinetics and drug dissolution. HSPiP software predicted various excipients based on HSP parameters to reduce the experimental screening duration and development stage. Cationic nanoemulsions may be a promising option for maximized drug access to the nasal cavity due to their small size (113 nm), high mucoadhesiveness (high positive zeta potential and mucoadhesive carbopol gel), and linoleic acid (as high content in the oil)-mediated drug permeation across the blood–brain barrier. Ex vivo permeation flux, the enhancement ratio, drug deposition, and the penetration property of CVE6 and CVE6 gel confirmed electrostatic and mucoadhesiveness worked in tandem for extended residence time in the nasal mucosa and, subsequently, augmented the drug’s access to the brain. Conclusively, this strategy is a promising and suitable alternative to conventional cream or oral tablets to control seizures with high therapeutic effectiveness and patient compliance.

## 4. Materials and Methods

### 4.1. Materials

Valproic acid sodium salt (VA, 98.0% pure) and polysorbate-80 were procured from Sigma Aldrich (Merck), Mumbai, Maharashtra, India). Soya lecithin powder (97%) was purchased from Otto Chemie Pvt. Ltd., Mumbai, India. HPLC (high-performance liquid chromatography)-grade solvents (methanol, ethanol, acetonitrile, and buffering reagents) were purchased from Merck, Mumbai, India. Edible safflower, flaxseed, and grape seed oils were purchased from a local medical shop. Buffer reagents (potassium dihydrogen phosphate, sodium chloride, and sodium hydroxide) were procured from S.D. Fine, Mumbai, India. In-house-distilled water was used as an aqueous solvent. For HPLC mobile phase preparation, Milli-Q water was used (Millipore, Burlington, MA, USA).

### 4.2. Methods

#### 4.2.1. Prediction and Simulation Study Using GastroPlus for Oral Tablet

The program was used to predict pharmacokinetic parameters (PK) of orally delivered VA tablets for adult patients with a dose of 250 mg. In the literature and on the DEPAKENE tablet label, varied bioavailability, absorption rate, and PK parameters have been described depending on the patient’s body weight. To avoid preclinical and clinical studies due to expensive and tedious investigations, the program assisted in predicting various PK parameters in a targeted patient for the desired dose, dosage form, dosage volume, and frequency of dosing frequency. For this, the program used three basic tabs such as (a) the compound tab, (b) the formulation tabs, and (c) the pharmacokinetic tabs. We used various literature data, experimental values, and by-default program-suggested values to run the simulation and prediction (as shown in Table 1). Moreover, parameter sensitivity assessment (PSA) was used to determine the impact of various factors (physicochemical properties of the drug and physiological conditions such as intestinal lumen and related factors) affecting the PK parameters of the drug. Physicochemical properties of the drug include the reference solubility, particle size, volume, density, logP, pKa, and molecular weight. Physiological factors include gastrointestinal pH, stomach volume, residence time, and radius. Formulation factors are the nanosize, shape, and solubility. The regional compartmental model predicts regional absorption of the drug through nine different GIT (gastrointestinal tract) sections (stomach, duodenum, ileum-1,2, jejunum-1,2, ascending colon, colon, and caecum). Total absorption indicates the sum of absorption from the GIT of patients. Prediction and simulation were carried out considering fast subjects to avoid a food interaction in the prediction model. The simulation time was 24 h for each run of prediction and simulation [25,26].

##### Hansen Solubility Parameters for VA and Excipients

Hansen solubility parameters have been used for various solvents, co-solvents, drugs, and human skin (normal and abnormal). The parameters were estimated using the HSPiP program. The fundamentals of the software are based on the physicochemical interactions (in terms of cohesive energy) of a solute for a particular solvent. These parameters are dispersion energy (δ_d_), polarity (δ_p_), and hydrogen bonding energy (δ_h_) [54,55]. Therefore, a solute interacts with a solvent through these cohesive forces working together. Thus, the difference in any parameter between a solute and solvent close to zero is considered miscible or soluble. Thus, the program predicted relevant excipients based on these HSP values of each excipient close to the HSP values of the model drug. The program estimated the values shown in Table 1. The HSP values of oils, lecithin, and PC were obtained from the literature, and these were calculated manually based on the percent composition of linoleic acid or phosphatidylcholine (PC) present [28].

##### Solubility of Valproate Sodium in Various Excipients

The solubility of VA was determined in various lipids, surfactants, and co-surfactants to identify the most suitable and biocompatible excipients for nasal nanoemulsion. Stearylamine was added to the organic phase to impose the cationic charge on a globular surface for adhesive purposes [13]. Flaxseed (50–70%), safflower (70–78%), and grape seed oil (70%) are prime sources of linoleic acid. Tween 80, Span-80, transcutol, propylene glycol, and lecithin were used as surfactants and co-surfactants. Briefly, a fixed amount of each excipient was transferred to a clean glass vial. A weighed amount of the drug was added to each vial containing the individual excipients. The glass vials were closed and sealed for the solubility study. The vial was placed inside a water shaker bath (Remi Shaker, Mumbai, India) set at a fixed temperature (40 °C) and shaking rate (75 rpm). The study was continued for 72 h to achieve equilibrium. Then, the mixture was centrifuged to obtain the supernatant liquid. The amount of the drug dissolved was assayed using a UV Vis spectrophotometer (U 1800, Japan) at 210 nm [10]. The study was repeated to obtain a mean and standard deviation (*n* = 3).

##### Pseudo Ternary Phase Diagram, Cationic Nanoemulsions, and Nanoemulsion Gel

To prepare a cationic nanoemulsion, a constant amount of stearylamine (0.1%) was used in the formulation. Based on HSP values and the experimental solubility of AV, excipients were selected. The excipient possessing HSP values close to the HSP values of AV and excipients with the highest solubility of AV were selected for cationic nanoemulsion. Thus, safflower seed oil, tween 80 + lecithin (1:1), and PG were selected as the oil, surfactant, and co-surfactant, respectively. To impose a cationic charge, a constant amount (0.1%) of SA (stearylamine as the cationic lipid) was incorporated into the organic phase of each formulation [34]. Various pseudoternary phase diagrams were constructed to identify the correct ratio of the surfactant to the co-surfactant (S_mix_). A slow and spontaneous titration method was adopted to prepare the nanoemulsion by varying the lipid-to-S_mix_ ratio [25]. A transparent and isotropic cationic nanoemulsion was selected for further characterization. To prepare a nanoemulsion gel, the cationic nanoemulsion was incorporated into a carbopol gel (1%). The final strength of the gel was 0.5% *w*/*w*. Each nanoemulsion and respective gel contained a constant amount of VA. For this, a weighed amount of carbopol 934 was dispersed into warm distilled water to obtain the final strength of 1% *w*/*w*. The dispersed gel was vigorously stirred using a mixer at high speed (10,000 rpm). The obtained gel was treated with a few drops (3–5 drops) of triethanolamine (base) as a cross-linking agent. The acidic solution of the carbopol dispersion was triggered for cross-linking under triethanolamine and become a transparent viscous gel. Equal weights of gel and lyophilized formulation were mixed together using a homogenizer to obtain a gel of 0.5% gel strength. The final concentration of AV in the gel product was approximately 5% *w*/*w*. The final pH of each formulation was adjusted to 6.8 to obtain good consistency and compatibility with nasal mucosa.

##### Thermodynamic Stability of Cationic Nanoemulsion: Freeze–Thaw Cycle and Ultracentrifugation

Each developed nanoemulsion was subjected to extreme physical (ultracentrifugation) and thermal stress (extreme low and extreme high temperatures). For this, each cationic nanoemulsion was stored in a clear glass vial, labelled, and sealed. Each formulation was separately stored in the stability chamber at the set temperature. A cycle of exposure to as low as freeze (−21 °C) and as high as thaw (40 °C) temperatures was repeated thrice followed by room temperature conditions. Each sample was withdrawn from both temperatures and kept at room temperature (25 °C) to resume its original stable form (isotropic liquid). In the second phase, each stable formulation was subjected to an ultracentrifugation step (22,000 rpm for 5 min). Any sign of physical instability (drug precipitation, color, creaming, and phase separation) was considered an unstable product and dropped out from further study. This freeze–thaw cycle was mandatory to identify the most stable product.

#### 4.2.2. Evaluation of Cationic Nanoemulsions and Gels

Nanoemulsions were characterized by globular size, size distribution, and zeta potential. These parameters were determined using a Zetasizer (Malvern Instrument Limited, Malvern, Worcestershire, UK). Formulations were diluted with distilled water before scanning for size analysis. In the case of zeta potential, the formulations were analyzed without dilution to obtain tangible zeta potential values. This value was expected to be positive for the cationic nanoemulsion, whereas the nanoemulsion without stearylamine was anticipated to be negative. The analysis was carried out at room temperature. The viscosity of each formulation was determined using a viscometer (Bohlin visco88, Malvern Instrument Ltd., Worcestershire, UK). The sample was processed at room temperature (25 °C). The study was replicated for the mean and standard deviation (*n* = 3). The values of pH were estimated using a calibrated digital pH meter.

##### Morphological Evaluation of the Optimized Cationic Nanoemulsion and Respective Gel

The optimized cationic nanoemulsion and respective gel were observed under cryogenic transmission electron microscopy (cryo-TEM) [56,57]. The tool was used to visualize the globular size, size distribution, and shape. For this, the sample was placed on a glass coverslip previously coated with poly-L-lysin (a fixative) [56]. Then, the sample was processed for cryogenic TEM by placing the sample on a copper screen with lacey carbon film and a blotting time of 5 s. Scanning was conducted using Thermofisher Krios G3 (Thermo Fisher Scientific India, Private Limited, Mumbai, India) equipment (low energy consumption method) coupled with a Bioquantum K3 detector [57,58]. The images were processed at an operating voltage power of 80 Kv (a Gatan cryoholder system) (Gatan, Inc. Corporate Headquarters 5794 W. Las Positas Blvd. Pleasanton, CA 94588, United States of America). Finally, the dried sample was scanned at various magnifications and resolutions. A fixed location was located and scanned for the sample. The process was conducted at room temperature. The wet sample was avoided in the scan due to poor scanning, and the resolution of images was observed by an interfered electronic beam.

##### Drug Content Estimation

The drug content was estimated from the optimized anionic nanoemulsion, cationic nanoemulsion, and respective gel formulations. In brief, a weighed amount of the formulation was dissolved in a methanol-chloroform mixture (1:10). The mixture was stirred for 10 min to extract the drug. The mixture was centrifuged for 15 min at 12,000 rpm to separate the low-density nanoemulsion from the insoluble drug and water. The supernatant and settled pellet were separately estimated to find the total drug and the entrapped drug. The drug was assayed using the validated HPLC method at 210 nm. The experiment was repeated to obtain the mean and standard deviation. In the case of gel formulation, a weighed amount of gel was dispersed into a water–ethanol mixture (1:2) to obtain the extracted drug. Then, the mixture was stirred for 15 min followed by centrifugation. The supernatant was used to estimate the drug content.

#### 4.2.3. In Vitro Drug Release Profile

The in vitro drug release profile for each nanoemulsion and the respective gel was determined using a dialysis membrane with a molecular weight cut-off of 12–14K Dalton (HiMedia, Mumbai, India). For this, a fixed dimension of the dialysis membrane was cut from ribbon and soaked in saline for 12 h before use. The activated dialysis bag was filled with the test sample and both ends were clipped with a plastic clipper. This maintained a constant effective surface area for drug release. The release medium (500 mL) was phosphate buffer at pH 6.8 and pH 7.4. A glass beaker containing the release medium was used for the drug release. The test sample bag was suspended in the release medium already placed on a heating magnetic stirrer. A Teflon-coated magnetic bead was used to maintain the temperature and uniform drug distribution within the bulk volume released from the bag. The sample was collated at different time points (0.5, 1, 2, 3, and 6 h). The withdrawn volume was replaced with the fresh-release medium. The withdrawn sample was filtered (0.22 µ as pore size) and used for the drug content released after each time point. The drug was analyzed using an HPLC method. The release medium chamber was maintained at a temperature of 32 ± 1 °C throughout the study. The effective surface area for passive diffusion of the drug was 1.34 cm^2^ functional at 32 ± 1 °C [36,59].

#### 4.2.4. Ex Vivo Drug Permeation and Drug Deposition Using a Goat Nasal Mucosa

Drug permeation and deposition studies were performed using an excised goat nasal mucosa obtained from a local slaughterhouse. The excised tissue was used 20 min after sacrifice to avoid tissue damage and death. The intact nose was obtained, and the skin was removed. Then, the nose was stored in a cold phosphate buffer solution (pH 7.4) [37]. The nasal mucosa was removed using surgical scissors and forceps without making any surgical cut in the desired area of the mucosal membrane. The obtained mucosal tissue was immersed in a freshly prepared Ringer’s solution with proper aeration [38]. The excised tissue has a dimension of 0.2 mm × 10 mm with an effective surface area of diffusion of 1.78 cm^2^. The tissue was mounted between the receptor and the donor chambers. The receptor chamber was filled with SNF (simulated nasal fluid) at pH 6.8 [40]. The release medium was maintained at 37 ± 1 °C by circulating hot water through a jacketed system around the chamber. A rice bead was placed inside the receptor chamber rotating at 300 rpm on a magnetic stirrer. A constant amount of the sample was placed on the mucosal adhesive side for drug permeation. Four groups were categorized as (a) CVE6-1, AVE6, CVE6-gel, and AVE6-gel. For comparison, the drug solution was used as a control in gel formulation. In each case, an equivalent amount of the drug was loaded on the effective permeation area. The sampling (1 mL using a syringe) was conducted at different time points such as 0.5, 1, 1.5, 2, 2.5, and 3 h. The withdrawn sample volume was replaced with an equal volume of the fresh medium. The sample was filtered using a membrane filter (0.2 µm) and the content of the drug was estimated using the HPLC method. The study was replicated to obtain the mean and standard values. The result was expressed as the percent of the drug permeated for the brain delivery or percent diffusion for the brain access or availability of the drug to the brain (ex vivo). The permeation parameters (steady state flux, targeted flux, permeability coefficient, and enhancement ratio) were estimated using the following equation:

J_ss_ = (dM/dt) × (1/A) = PC (2)

where A, P, and C represent the effective surface area for diffusion, the permeability diffusion coefficient, and the initial loaded content of the drug, respectively. Jss indicates the steady state flux of the solution as per Fick diffusion in Equation (2). The value of dM indicates the amount of the drug diffused across the mucosal membrane within a given time point (dt). The study was conducted for up to 360 min to avoid the loss of the natural integrity of tissue and tissue viability [39].

A drug deposition study was conducted after the completion of the ex vivo permeation study. The mounted tissue was removed from the diffusion cell. Each tissue was separately sliced into small pieces. The sliced pieces were transferred to a vial containing methanol and chloroform (1:2). The mixture was stirred for 12 h under closed conditions using a magnetic bead. The drug was extracted from the tissue and subjected to centrifugation. The fatty debris and tissues settled at the bottom as pellets and the supernatant (clear solution) was removed for the drug analysis. The supernatant was filtered using a membrane filter and analyzed using the HPLC method [10,60].

#### 4.2.5. Confocal Laser Scanning Microscopy (CLSM)

To visualize the degree of drug penetration, the same formulations and control were reformulated using rhodamine 123 as a probe in the formulation. The composition and experimental conditions were kept constant as in the ex vivo permeation and drug deposition study section. The dye was present as 0.01% *w*/*v* in each formulation. The Franz diffusion cell, tissue mounting, release SNF, volume, pH, and loaded dose were constant for 3 h. After 3 h of permeation study, the tissue was removed for each group (five groups), and the adhered material was washed with running water. The tissue was sliced as per CLSM requirements. The treated and untreated skin was sliced into small pieces using a microtome. The tissue specimen was placed on the glass coverslip and air-dried for 12 h. Each tissue was visualized under CLSM and evaluated for globular penetration across the mucosal membrane (Fluorescence Correlation Microscope-Olympus FluoView FV1000, Olympus, Melville, NY, USA) with an argon laser beam with excitation at 488 nm and emission at 590 nm [15,61].

## Figures and Tables

**Table 1:** Summary of input data for GastroPlus simulation and prediction of VA sodium.

Parameter	Values
Molecular formula	C_8_H_15_NaO_2_
Molecular weight (g/mol)	166.19
Melting point (°C)	300
Aqueous solubility (mg/mL) at 25 °C	<1
Density (g/mL)	0.9
Pka	5.14
Log p	3.08
Apparent permeability coefficient (cm/h) across hCMEC/D3 and CC-2565 of in vitro blood brain barrier	0.625
Dose (mg)	200
Body weight (kg)	70
Dosing volume (mL)	1
Mean precipitation time (s)	30
AUC (µg. h/mL)	10–160
C_max_ (mg/L)	~120
T_max_ (h) (mean)	5
Elimination half-life (h)	8–15
Clearance (L/h)	0.206–1.154
Plasma protein binding (%)	90–95
V_d_ (L)	8.4–23.3
pH for reference solubility	7.0
Simulation time (h)	24

**Table 2:** Summary of HSP values for the drug and selected excipients.

Drug and Excipient	δ_d_ (MPa^1/2^)	δ_p_ (MPa^1/2^)	δ_h_ (MPa^1/2^)
AV	16.1	4.3	9.0
Safflower seed oil (87%) *	14.5	2.7	5.3
Grape seed oil (70%) *	11.69	2.17	4.27
Flaxseed oil (60%) *	10.02	1.86	3.66
Tween 80	16.6	5.3	7.5
Span 80	16.7	6.1	12.4
Lecithin (PC as 20%) *	3.2	0.54	0.64
Linoleic acid *	16.7	3.1	6.1
Transcutol HP	16.0	2.8	6.2
PG ^ϕ^	16.8	10.4	21.3
PC *	16	2.7	3.2

* Estimated using reference [28]; ^ϕ^ [30].

**Table 3:** Summary of selected cationic/anionic VA-loaded nanoemulsions and their evaluated parameters.

Code	SO (%)	S_mix_ (%)	Water (%)	S_mix_ Ratio	ST (%)	Size (nm)	PDI	ZP (mV)	%T	Product Strength (% *w*/*w*)
CVE1	16.46	30.21	48.59	1:2	0.04	162	0.27	+24.7	98.5	0.4
CVE2	20.75	23.5	50.22	1:3	0.05	189	0.32	+26.8	96.8	0.5
CVE3	14.72	21.67	57.12	1:2	0.06	185	0.31	+31.6	97.2	0.6
CVE4	19.88	43.51	32.16	1:2	0.04	148	0.18	+23.9	96.9	0.4
CVE5	9.8	21.84	63.04	2:1	0.05	79	0.11	+27.1	95.3	0.5
CVE6	14.46	17.15	60.99	3:1	0.07	113	0.26	+34.7	97.8	0.7
AVE6	14.46	17.15	60.92	3:1	0.0	126	0.29	−22.8	95.6	0.7
	Nanoemulsion gel (0.5% *w*/*w*) composition (VA strength)					Evaluated parameters				
0.5% VE gel	NE (g)	Gel-blank (g)	Triethanolamine (g)	CVE6:gel ratio		Size (nm)	PDI	ZP (mV)	Viscosity (cP)	pH
CVE6 gel (0.35%)	1	0.95	0.05 g	1:1		129	0.24	+21.9	1837.3	6.8
AVE6 gel (0.35%)	1	0.95	0.05 g	1:1		142	0.31	−26.5	1907.1	7.1

Note: SO = Safflower, S_mix_ = Tween 80-lecithin: transcutol, ST = stearylamine (cationic charge inducer), PDI = Polydispersity index; ZP = zeta potential, NE = Nanoemulsion, CVE = Cationic NE, VE = Valproic acid loaded nanoemulsion, AVE6 = Anionic valproic acid loaded nanoemulsion.

**Table 4:** Freeze–thaw and centrifugation cycles and observation.

Formulations	Freezing (−21 °C)	Room Temperature	Thaw (40 °C)	Centrifugation	Inference *
CVE1	✓	✓	✓	✓	Stable
CVE2	✓	✓	✓	✓	Stable
CVE3	✓	✓	✓	✓	Stable
CVE4	✓	✓	✓	✓	Stable
CVE5	✓	✓	✓	✓	Stable
CVE6	✓	✓	✓	✓	Stable
AVE6	✓	✓	✓	✓	Stable

Note: * Recovery of original form/state of nanoemulsion at room temperature after exposure to the extreme temperature was considered stable in inference. Formulations exhibiting any signs of instability in terms of drug precipitation, phase separation, color development, and creaming were dropped out from further studies. The symbol “✓” means passed the test.

## Data Availability

Not applicable.

## Supplementary Materials

The following supporting information can be downloaded at: https://www.mdpi.com/article/10.3390/gels9080603/s1, Figure S1: Representative images of cryo-TEM micrographs: (A) CVE6 nanoemulsion, (B) AVE6 nanoemulsion, (C) CVE6 gel, and (D) AVE6 gel. Magnification 49000X.
