# Supplementary material for: GastroPlus- and HSPiP-Oriented Predictive Parameters as the Basis of Valproic Acid-Loaded Mucoadhesive Cationic Nanoemulsion Gel for Improved Nose-to-Brain Delivery to Control Convulsion in Humans
Source: Gels. 2023 Jul 26;9(8):603. doi: 10.3390/gels9080603 (PMC10453491; doi:10.3390/gels9080603)
Supplement: Supplementary file 1 [file gels-09-00603-s001.zip › gels-2466676-supplementary.pdf]

gels

Supplementary Materials

GastroPlus- and HSPiP-Oriented Predictive Parameters as the Basis of Valproic Acid-Loaded Mucoadhesive Cationic Nanoemulsion Gel for Improved Nose-to-Brain Delivery to Control Convulsion in Humans

Afzal Hussain [1,*], Mohammad A. Altamimi [1], Mohhammad Ramzan [2], Mohd Aamir Mirza [3] and Tahir Khuroo [4]

[1] Department of Pharmaceutics, College of Pharmacy, King Saud University, Riyadh 11451, Saudi Arabia; maltamimi@ksu.edu.sa
[2] School of Pharmaceutical Sciences, Lovely Professional University, Phagwara 144411, India; mohhammad.26652@lpu.co.in
[3] Department of Pharmaceutics, School of Pharmaceutical Education and Research, Jamia Hamdard, New Delhi 110062, India; aamir.mirza@jamiahamdard.ac.in
[4] PGx Global Foundation, Houston, TX 77035, USA; tahir@pgxglobal.org
* Correspondence: amohammed2@ksu.edu.sa

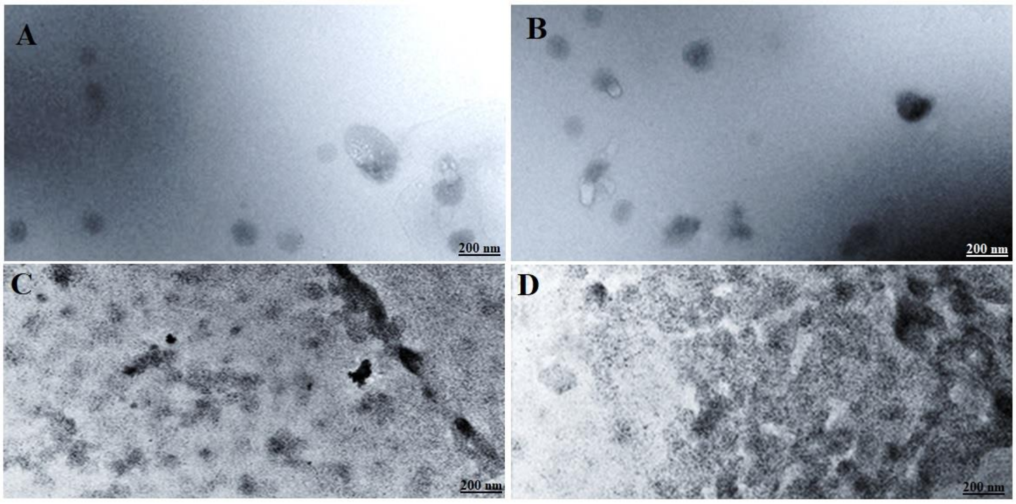

Figure S1. Representative images of cryo-TEM micrographs: (A) CVE6 nanoemulsion, (B) AVE6 nanoemulsion, (C) CVE6 gel, and (D) AVE6 gel. Magnification 49000X.
